# Modelling single nucleotide effects in *phosphoglucose isomerase* on dispersal in the Glanville fritillary butterfly: coupling of ecological and evolutionary dynamics

**DOI:** 10.1098/rstb.2009.0005

**Published:** 2009-06-12

**Authors:** Chaozhi Zheng, Otso Ovaskainen, Ilkka Hanski

**Affiliations:** Department of Biological and Environmental Sciences, University of HelsinkiPO Box 65, 00014 Helsinki, Finland

**Keywords:** evolution of dispersal, life-history evolution, metapopulation, correlated random walk, *phosphoglucose isomerase* gene, habitat fragmentation

## Abstract

Dispersal comprises a complex life-history syndrome that influences the demographic dynamics of especially those species that live in fragmented landscapes, the structure of which may in turn be expected to impose selection on dispersal. We have constructed an individual-based evolutionary sexual model of dispersal for species occurring as metapopulations in habitat patch networks. The model assumes correlated random walk dispersal with edge-mediated behaviour (habitat selection) and spatially correlated stochastic local dynamics. The model is parametrized with extensive data for the Glanville fritillary butterfly. Based on empirical results for a single nucleotide polymorphism (SNP) in the *phosphoglucose isomerase* (*Pgi*) gene, we assume that dispersal rate in the landscape matrix, fecundity and survival are affected by a locus with two alleles, A and C, individuals with the C allele being more mobile. The model was successfully tested with two independent empirical datasets on spatial variation in *Pgi* allele frequency. First, at the level of local populations, the frequency of the C allele is the highest in newly established isolated populations and the lowest in old isolated populations. Second, at the level of sub-networks with dissimilar numbers and connectivities of patches, the frequency of C increases with decreasing network size and hence with decreasing average metapopulation size. The frequency of C is the highest in landscapes where local extinction risk is high and where there are abundant opportunities to establish new populations. Our results indicate that the strength of the coupling of the ecological and evolutionary dynamics depends on the spatial scale and is asymmetric, demographic dynamics having a greater immediate impact on genetic dynamics than vice versa.

## 1. Introduction

Dispersal comprises a complex life-history syndrome that especially influences the demographic dynamics of species in temporally ephemeral and spatially fragmented habitats (Olivieri & Gouyon 1997; Hanski 1999; Ronce 2007; Moore & Hendry 2009). On the other hand, demographic dynamics under the prevailing environmental conditions can be expected to influence selection on dispersal. Identifying the evolutionarily stable dispersal strategy under particular environmental conditions is a classic topic in life-history theory (Hamilton & May 1977; Comins *et al*. 1980; Ronce & Olivieri 2004). These considerations also have great practical significance. Especially in the past decades and centuries, humans have greatly modified many environments on the Earth, typically causing loss and fragmentation of natural habitats. Increasing fragmentation of habitats may select either for an increase (Heino & Hanski 2001) or a decrease in dispersal rate (Cody & Overton 1996; Cheptou *et al*. 2008), depending on a number of factors but especially on the cost and benefit of dispersal in terms of mortality and the chance of locating a new favourable patch of habitat for reproduction (Heino & Hanski 2001).

Evolutionary models of dispersal do not usually specify the actual genetic mechanism on specific traits that would affect dispersal, but the models typically employ the quantitative genetics framework and assume a large number of genes with a small effect (Ronce & Olivieri 2004). In a few cases, it is known that some component of dispersal is affected by a single locus. For instance, wing polymorphism in some insect species is thought to be controlled by a single gene with two alleles (Roff 1986). One example of a candidate gene is the *phosphoglucose isomerase* (*Pgi*) gene in butterflies (Watt 1983, 1992; Haag *et al*. 2005), a willow beetle (Rank *et al*. 2007) and probably many other invertebrates. In the Glanville fritillary butterfly (*Melitaea cinxia*), molecular variation in *Pgi* is associated with dispersal rate in the field (Niitepõld *et al*. in press) and in a large outdoor population cage (Saastamoinen & Hanski 2008), at least partly because of association between dispersal rate and flight metabolic rate (Haag *et al*. 2005; Niitepõld *et al*. in press). Saastamoinen (2008) has shown that mobility in the population cage is heritable, and in the field there is spatial variation in the dispersal rate between individuals originating from newly established versus old local populations (Hanski *et al*. 2002, 2004), which is most parsimoniously explained by heritable variation in dispersal. Molecular variation in *Pgi* is also correlated with variation in other life-history traits, such as egg clutch size (Saastamoinen 2007*a*) and even with the growth rate of small local populations (Hanski & Saccheri 2006).

The purpose of the present study is to construct and analyse an evolutionary, individual-based model of dispersal that is appropriate for the Glanville fritillary and other comparable species living in fragmented landscapes, implementing the genetic architecture that is relevant for *Pgi*. Our particular aim is to analyse selection that is imposed by the spatial configuration of the habitat at the landscape level and thereby to predict patterns in the spatial distribution of the *Pgi* genotypes and dispersal phenotypes within and among different kinds of landscapes. This study was motivated by the availability of extensive empirical data for the Glanville fritillary from a large network of habitat patches in the Åland Islands in southwest Finland, where there is much spatial variation in patch density and other properties of the landscape (Hanski 1999; Nieminen *et al*. 2004). Apart from examining how the structure of the fragmented landscape influences the evolution of dispersal in metapopulations, we analyse the possible coupling between the demographic (ecological) and genetic (evolutionary) dynamics involving dispersal.

## 2. Empirical data

The metapopulation of the Glanville fritillary butterfly in the Ålands Islands in southwest Finland has been studied since 1991 (Hanski 1999). These studies have produced much empirical data on dispersal, local population dynamics, metapopulation dynamics and spatial genetic structure. The structure of the landscape has been described by Hanski *et al*. (1996), Moilanen & Hanski (1998), Hanski (1999) and Nieminen *et al*. (2004). Within an area of 50 by 70 km, there are altogether approximately 4000 discrete and mostly very small dry meadow habitat patches with an average area of 0.15 ha. Suitable habitat is largely defined by the presence of one or both of the two larval host plant species, *Plantago lanceolata* and *Veronica spicata*.

The key empirical data on which the present modelling is based and with which the model predictions are compared are as follows.

### (a) Life cycle and reproduction

There is one generation per year. The adults eclose in early June and live for an average of one to two weeks depending on weather conditions. Most females mate soon after eclosion in the natal population, but 10–30 per cent of females mate a second time, based on experiments in a large outdoor cage (Sarhan & Kokko 2007; M. Saastamoinen 2007, personal communication). Re-mating may happen either in the natal population or in another population following dispersal. Females lay large batches of 50–300 eggs at intervals of one or more days, depending on weather conditions (Saastamoinen 2007*a*). Each egg batch develops into a larval group, which remains as a group until the spring in the following year. For more details on the life history, see Hanski (1999). Empirical results on mating and oviposition have been reported by Wahlberg (1995), Boggs & Nieminen (2004) and Saastamoinen (2007*a*,*b*).

### (b) Demographic dynamics

The Glanville fritillary metapopulation has been surveyed annually since 1993. The survey is conducted in late summer prior to larval diapause. The larval groups are highly visible due to a silken web spun by the larvae around the host plant. In these surveys, all, approximately 4000, meadows are visited by field assistants and the numbers of larval webs are counted. The detection probability of larval groups has been estimated to be approximately 0.5 (Nieminen *et al*. 2004).

These data allow the calculation of several statistics describing the demographic dynamics and patterns, which can be compared with simulation results: the distribution of local population sizes in terms of the number of larval groups in late summer; the average fraction of habitat patches occupied in 1 year and variation among the years; and the numbers of local extinctions and re-establishment of new populations per year. We have used Hanski *et al*.'s (1996) classification of the entire 4000 patch network into sub-networks with dynamically semi-independent metapopulations. These sub-networks vary in terms of the number, mean area and spatial connectivity of the constituent habitat patches and thus allow informative comparisons between landscapes with different properties. Hanski (1999), Nieminen *et al*. (2004) and Hanski & Meyke (2005) review results on local and metapopulation dynamics.

### (c) Dispersal

Mark–recapture studies on dispersal are reported by Hanski *et al*. (1994) and Kuussaari *et al*. (1996), while Hanski *et al*. (2000), Ovaskainen (2004) and Ovaskainen *et al*. (2008*b*) describe statistical modelling of dispersal. Other biological results on dispersal have been reported by Hanski *et al*. (1995) and Kuussaari *et al*. (1996).

### (d) Spatial genetic patterns

Saccheri *et al*. (2004) used four allozymes and two microsatellite markers to examine population differentiation across the Åland Islands using pairwise *F*_ST_. Orsini *et al*. (2008) showed that the spatial genetic structure correlates better with the past than the present spatial demographic structure.

### (e) Contrast between newly established versus old local populations

Hanski *et al*. (2002) and Ovaskainen *et al*. (2008*c*) have showed that female butterflies originating from isolated local populations that had been established by the mothers of the focal individuals are more dispersive than females originating from isolated old local populations. This result implies higher than average dispersal rate among individuals that establish new populations at isolated habitat patches, high heritability of the relevant traits of dispersal and rapid loss of the more dispersive individuals from the new populations in subsequent years. Further studies have demonstrated that females in isolated new populations have higher than average flight metabolic rate (Haag *et al*. 2005), and that flight metabolic rate is associated with dispersal rate in the field (Niitepõld *et al*. in press). Some other life-history traits, including egg clutch size, exhibit differences between newly established versus old populations (Saastamoinen 2007*b*).

### (f) *Phosphoglucose isomerase* locus

A series of field and laboratory studies has documented strong and consistent associations between molecular variation in the *Pgi* locus and variation in flight metabolic rate (Haag *et al*. 2005), dispersal rate in the field (Niitepõld *et al*. in press), body temperature in low ambient temperatures (Saastamoinen & Hanski 2008), egg clutch size (Saastamoinen 2007*a*), lifespan (Saastamoinen *et al*. 2009) and local population growth rate (Haag *et al*. 2005; Hanski & Saccheri 2006). Orsini *et al*. (2009) have identified a single nucleotide polymorphism (SNP) at *Pgi*, AA111, which accounts for the functionally significant variation in *Pgi*.

For this paper, we have re-analysed the data used by Hanski & Saccheri (2006) on the *Pgi* allelic composition in local populations. We use the above-mentioned sub-networks of habitat patches (Hanski *et al*. 1996) to test model predictions about the influence of landscape structure on *Pgi* allelic composition in the respective sub-networks.

## 3. The individual-based model

We construct an individual-based sexual model that corresponds to the life cycle of the Glanville fritillary (for a related asexual model, see Hanski *et al*. 2004). The dispersal model (§3*a*) assumes that the radii of the habitat patches are substantially less than the interpatch distances. We have therefore merged nearby habitat patches in the real network into single patches, with the area equalling the pooled area of the original patches and the location of the new patch given by the area-weighted average of the locations of the original patches. Following the merging of the patches, we ended up with a network of 2254 patches. This merging of the patches does not affect the clustering of patches into the sub-networks as described above. In all analyses, we simulated the dynamics of the system in the entire network, even if the results were recorded for individual sub-networks.

For the purpose of model construction, we split the life cycle into (adult) dispersal phase and local (larval) dynamics, which are described in turn below, followed by a description of how the genetic dynamics were modelled.

### (a) Dispersal

We assume that all adults eclose simultaneously in the beginning of the flight season and that they disperse independently of each other (no density dependence at the adult stage). Butterflies are assumed to obey correlated random walk both within habitat patches and while moving in the surrounding landscape matrix, where there are no host plants for oviposition. An important component of the movement model is edge-mediated behaviour (habitat selection), meaning that movements are biased towards the habitat patch when the individual is located close to the patch boundary. For mathematical convenience, we employ a diffusion approximation of the random walk model (Turchin 1998; Ovaskainen & Cornell 2003). The diffusion model has been shown to fit well mark–recapture data for the Glanville fritillary and other butterflies (Ovaskainen *et al*. 2008*a*,*b*).

The parameters of the diffusion model are the diffusion coefficients *D*_p_ in habitat patches and *D*_m_ in the matrix, the respective mortality rates *μ*_p_ and *μ*_m_, and the coefficient *k*_p_, which gives the relative density (number per unit area) of individuals in the habitat patches over the density in the matrix (by definition, matrix preference is *k*_m_=1). Under the assumption that patch sizes are small compared with interpatch distances, movement probabilities between the patches and the times that the individuals are expected to spend in the patches and in the matrix can be computed analytically (Ovaskainen & Cornell 2003; Zheng *et al*. in press). As described in the electronic supplementary material, this allows fast simulation of movements. As butterflies tend to spend some time in the natal patch before commencing subsequent movement behaviour (Ovaskainen *et al*. 2008*b*), we assume that butterflies stay in the natal patch *t*_c_ days (or less if they die) before starting to obey the dispersal model.

### (b) Mating and oviposition

The dispersal model generates a movement track for each individual in continuous time. To simplify the simulation of matings and ovipositions that take place during the dispersal phase, we discretize the movement tracks to the resolution of Δ*t*=0.1 days. We assume that a female mates during one time-step with probability *p*_M_=*η*[1−exp(−*λ*_M_*m*Δ*t*)], where *m* denotes the number of males in the habitat patch in which the focal female is currently located. Parameter *η* represents the tendency of mating, with *η*=1 for unmated females and *η*=*η*_0_ for females that have already mated at least once. Repeated mating in the same day does not occur (M. Saastamoinen, personal communication); hence we assume that *p*_M_=0 if the female has already mated in the current day. The mate for the female is chosen randomly among the *m* available males.

A mated female is assumed to lay an egg clutch with probability *p*_L_=1−exp(−*λ*_L_Δ*t*), with the exception that *p*_L_=0 if the female is located in the matrix or she has laid in the current day. In the case of multiple matings, the father of an egg clutch is the last male to mate with the female (Sarhan 2006).

### (c) Larval survival

Each egg clutch in patch *i* is assumed to develop into a larval group in the autumn and to survive over the winter with probability *p*_*i*_. To simulate the effects of environmental and regional stochasticities, we randomize (independently for each year) logit(*p*_*i*_) from a multinormal distribution with mean *μ*_s_**1** and variance–covariance matrix **Σ**, where $\sum_{ij}=\sigma_{\text{S}}^{2}\;\text{exp}\;(-d_{ij}/d_{\text{c}})$ and *d*_*ij*_ is the distance between patches *i* and *j*. The parameter *d*_c_ represents the average spatial autocorrelation distance. Additionally, we assume that all egg groups in a given patch may die simultaneously (due to a local catastrophe) with probability *e*_*p*_.

For the groups that survive, local density dependence is assumed to operate via competition for food in the late larval instars (Hanski 1999; Nieminen *et al*. 2004). We assume that the number of adults eclosing from each surviving larval group in patch *i* follows the Poisson distribution with mean $\bar{n}=(n_{0}/1+b_{i}n_{0}/K_{i})$, where *b*_*i*_ denotes the number of larval groups that survived over the winter; *K*_*i*_ is the carrying capacity of patch *i*; and *n*_0_ is the number of adults that would eclose per larval group in the absence of density dependence. We assume that the carrying capacity scales with the area of patch *i* as $K_{i}=n_{0}c_{0}A_{i}^{c_{1}}$, where *A*_*i*_ is the area of patch *i* in ha, and the product of *n*_0_ and *c*_0_ gives the carrying capacity in a patch with the area of 1 ha.

### (d) Genetic dynamics

To examine the spatial genetic structure predicted by the model, we simulated *L*=6 independent neutral loci, four of which are assumed to be allozymes and two microsatellite loci. These markers were assumed because they match what Saccheri *et al*. (2004) have studied in an empirical study. We assume *N*_*l*_ (*l*=1, …, *L*) alleles in each locus, setting the *N*_*l*_'s at the values observed by Saccheri *et al*. (2004). We assume a *k*-allele model, each allele mutating into one of the other alleles with probability *u* in each generation.

To model the influence of molecular variation at *Pgi* on dispersal, we assume that dispersal rate in the matrix, given by parameter *D*_m_, is determined by one locus with two alleles, A and C. This corresponds to the two nucleoties in the *Pgi* SNP AA111 (§2). We assume that individuals with the C allele, the AC heterozygotes and the CC homozygotes, have higher mobility than the AA homozygotes. More precisely, we assume that the flight bouts in the matrix are longer for the more mobile individuals, but that there is no difference in the behaviour in the patches (for justification and alternative assumptions, see the electronic supplementary material, figures S1–S3). In the diffusion model, the mean displacement that an individual is expected to move in a homogeneous environment within its lifetime is given by $E[r]=(\pi /2)\sqrt{D/\mu }$ (Turchin 1998), where *D* is the dispersal rate and *μ* is the mortality rate. Assuming that the environment would consist solely of the matrix habitat, the parameters in table 1 give *E*[*r*]=940 m for the AA individuals and *E*[*r*]=9400 m for the AC and CC individuals. However, while moving in a real patch network, the movements in the matrix are interrupted by visits to the patches, reducing the expected lifetime movement range (see the electronic supplementary material, figure S4).

Based on empirical results, we assume two other life-history differences between the *Pgi* genotypes. First, AC heterozygote females have 20 per cent greater egg clutch size than females of the other genotypes (Saastamoinen 2007*a*). Second, the CC homozygotes have very low early life survival (Orsini *et al*. 2009). We have assumed that only 10 per cent of the CC homozygotes survive to eclosion. Assuming a large panmictic population, these two assumptions lead to the maintenance of *Pgi* polymorphism, with the equilibrium frequency of the C allele at 0.08 (see the electronic supplementary material).

### (e) Parameter values

Constructing and parametrizing an individual-based evolutionary metapopulation model is challenging, made even more so by the intrinsic difficulty of realistic modelling of dispersal behaviour in a heterogeneous landscape. Although there is a large amount of information available for the Glanville fritillary metapopulation from the Åland Islands, there are still critical data lacking for some key processes, such as local density dependence. Nonetheless, many parameters can be estimated with independent data (table 1; see the electronic supplementary material for justification). We assume constant values for these parameters throughout the paper. The remaining parameters that could not be estimated independently were adjusted to match the simulated demographic dynamics with the observed demographic dynamics. The demographic model cannot therefore be critically tested with independent data, but this model provides a realistic framework to analyse the genetic dynamics of the *Pgi* locus and the evolutionary dynamics of dispersal. Predictions concerning the spatial variation in the *Pgi* allelic composition among individual populations and among independent metapopulations occupying separate sub-networks of habitat patches can be tested with independent data.

### (f) Resolving the coupling of genetic and demographic dynamics

To examine the causality in the coupling of demographic (ecological) and genetic (evolutionary) dynamics, we performed additional simulation experiments for hypothetical networks consisting of identical habitat patches arranged as a 10×10 regular lattice. We varied both the density of the patches and the size of the patches to assess the effect of network structure on the dynamics. In addition to recording the relationships between network structure, metapopulation size and the frequency of the C allele, we conducted a set of perturbation experiments. We first sampled a set of 100 snapshots from the stationary state of the model to be used as initial conditions. We then perturbed in the initial conditions as follows. In demographic perturbations, the size of the local populations was either doubled (by making a copy of each larval group) or halved (by removing each larval group with probability 0.5). In genetic perturbations, the frequency of the C allele was either increased (by transforming AA individuals to AC individuals with probability 0.5) or decreased (by transforming AC to AA with probability 0.5). We then followed the dynamics of the perturbed (treatment) and the unperturbed (control) metapopulations for 1 year, averaging over 100 replicates for each initial condition to remove the effect of demographic noise. We used the same values for the survival probability of larval groups and the same realizations of the probability *e*_*p*_ of catastrophic local extinction for each pair of control and treatment to remove the effect of environmental noise. We measured the demographic and genetic responses by computing the difference between the perturbed and unperturbed metapopulations in the growth rate and in the frequency of the C allele.

## 4. Results

### (a) Demographic and neutral genetic dynamics

**Figure 1:**
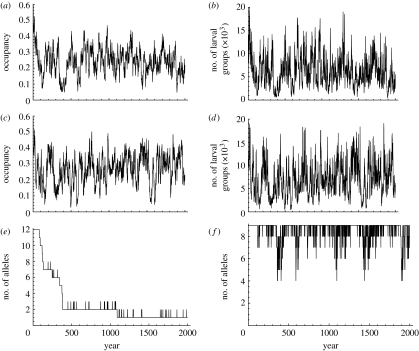
(*a*,*b*) Simulated time-series of metapopulation dynamics in a monomorphic population. (*a*) The fraction of occupied habitat patches and (*b*) the pooled number of larval groups in the metapopulation in the autumn before diapause are given. (*c*,*d*) Same as that in (*a*,*b*), but now for a metapopulation that is polymorphic for the *Pgi* gene. (*e*,*f*) The corresponding time-series for the number of alleles in one allozyme and in one microsatellite in the monomorphic metapopulation is given. Simulations are based on the parameter values shown in table 1.

**Figure 2:**
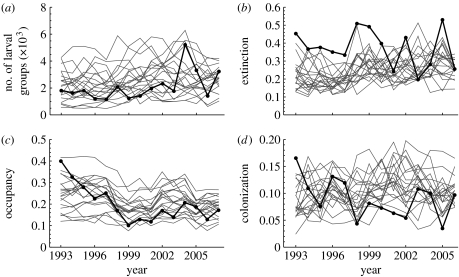
Comparison between observed and simulated time-series in the entire metapopulation. (*a*) The number of larval groups in the autumn, (*b*) the extinction rate, (*c*) the fraction of occupied patches and (*d*) the colonization rate are given. The thick curves depict real data and the thin curves depict independent 15-year samples of the simulations at the stationary state (initial 500 years dropped as a transient, different samples separated by 75 years). The simulated data were sampled in the same way as the real data, by including only those habitat patches that were surveyed in the corresponding year. Based on data from Nieminen *et al*. (2004), we assumed in the simulation that the probability of observing a larval group is 0.5, and that the probability of recording an occupied patch as empty is 0.1. Parameter values are as given in table 1 for a monomorphic population.

To match the predicted and observed average size of the metapopulation in terms of the number of occupied meadows and the pooled number of larval groups in the autumn, we adjusted two parameters in table 1: *μ*_s_ and *n*_0_. Similarly, to set the corresponding annual variation in metapopulation size, we adjusted the standard deviation of the survival of larval groups over winter (*σ*_s_). The predicted time-series are shown in figure 1*a*,*b*. Figure 2 compares the observed time-series for 15 years with a set of 20 independent comparable predicted series, indicating that the model reproduces the observed dynamics of the entire metapopulation reasonably well. The predicted annual population turnover rate, consisting of local extinctions and re-colonizations of currently empty patches, is similar to the observed turnover rate in the real data (figure 2). The annual rate of extinction is approximately 30 per cent and the rate of re-colonization of unoccupied patches is approximately 10 per cent. Turnover mostly affects the smallest local population.

We examined the sensitivity of the predicted dynamics to changes in two key parameters of dispersal and local dynamics, which greatly influence the rate of establishment of new populations and the rate of local extinction. To do this, we repeated simulations for three values of the diffusion rate in the matrix (*D*_m_) and three values of the average survival rate of larval groups over winter (*μ*_s_) (table 2). These values include the diffusion rates considered to be realistic for the different *Pgi* genotypes, while the survival values bracket the default value in table 1. As expected, the average size of the metapopulation is very sensitive to winter survival (table 2). The size of the metapopulation also increases with the diffusion rate *D*_m_. One critical assumption here is the relationship between *D*_m_ and parameter *k*_p_, which sets the strength of habitat selection (the density of individuals in the habitat patches over the density in the matrix; Ovaskainen 2004). We have assumed that *k*_p_ increases in proportion to the square root of *D*_m_. The justification of this assumption and two alternative assumptions are discussed in the electronic supplementary material (figures S1–S3).

**Figure 3:**
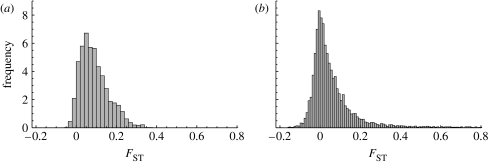
Histogram of *F*_ST_ values for all pairs of local populations. (*a*) Empirical results for 1995 (from Saccheri *et al*. 2004) and (*b*) a snapshot from the simulation without *Pgi* polymorphism are shown. *F*_ST_ was estimated according to Weir & Cockerham (1984).

Assuming the set of genetic markers used in the empirical study by Saccheri *et al*. (2004) and the parameter values in table 1 and figure 1*e*,*f* shows the predicted molecular diversity for one allozyme and one microsatellite marker. Given the mutation rates in table 1, the allozymes have one or two alleles at quasi-equilibrium, while the microsatellites have from approximately *five* alleles up to the observed number of alleles (table 1; figure 1). The main discrepancy is the much lower molecular diversity in the allozymes than what was observed empirically for some of them, e.g. for *pep A* with 12 alleles (Saccheri *et al*. 2004). It is possible that the mutation rate assumed for allozymes was too small. Another possibility is that some allozymes are not neutral and that selection maintains high allelic diversity. This is most likely so for *Pgi* (§4*b*; not included as a neutral locus in the simulation), for which there were eight alleles in the dataset analysed by Saccheri *et al*. (2004). Figure 3 shows that the predicted isolation by distance relationship characterized by the distribution of pairwise *F*_ST_ values among populations matches well with the observed result. This indicates that the assumptions made on the demographic dynamics are consistent with the observed neutral genetic dynamics, and thus the model provides a well-justified baseline for examining non-neutral dynamics.

### (b) Comparison between predicted and observed spatial patterns in the *Pgi* allele frequency

We next introduce a locus with two alleles, corresponding to the *Pgi* locus that has been studied empirically (§2). The parameter values assumed for the different *Pgi* genotypes (table 1) are described in §3*e*. We start by comparing the predicted spatial variation in the *Pgi* allele frequency with empirical data. In §4*c* we analyse the model further and examine other predicted consequences of *Pgi* polymorphism for the spatial and temporal dynamics of the metapopulation.

The model predictions that are testable with independent empirical data concern the frequency of the C allele in local populations of dissimilar ages and spatial connectivities, and in sub-networks of habitat patches with different numbers, average sizes and average connectivities. As a measure of population dynamic connectivity of patch *i* in year *t*, we use $S_{i}(t)=\sum_{j\neq i}N_{j}(t-1)\;\text{exp}\;(-d_{ij})$, where *N*_*j*_(*t*−1) is the number of larval groups in patch *j* in the previous year and *d*_*ij*_ is the distance (km) between patches *i* and *j*.

**Figure 4:**
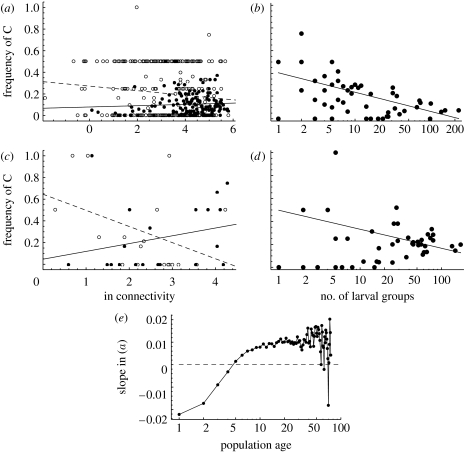
Comparison between predicted and observed spatial variations in the frequency of the C allele among local populations. (*a*,*c*) The frequency of C as a function of connectivity in newly established (open circles, dashed regression lines) and old populations (filled circles, solid regression lines) is given. (*b*,*d*) One snapshot of the frequency of C in sub-networks of habitat patches as a function of the pooled number of larval groups in the network at the time of sampling is shown. In the regression lines, the networks in which the C allele was absent (frequency 0) have been excluded. (*a*,*b*) Model predictions, (*c*) the empirical result from fig. 2*b* in Haag *et al*. (2005) and (*d*) an empirical result calculated with the data described by Hanski & Saccheri (2006) are shown. (*e*) How the slope in (*a*) depends on the age of the population (years since the population has been established), with data for 1500 independent snapshots is shown.

First, we compare the frequency of C in newly established versus old local populations. Consistent with previous empirical work (§2), the newly established populations were colonized in the previous generation, while, by definition, the old populations had persisted more than 5 years since the colonization. Figure 4*a* gives the predicted result for one snapshot from the stationary state of the model. In this snapshot, the frequency of C decreases with increasing connectivity in newly established populations, but increases with connectivity in old populations. Examining the slope of the frequency of C against connectivity in 150 independent snapshots shows that the slope in the simulations was higher for old than that for the newly established populations with probability 0.96. The mean slope increases systematically with population age and turns from negative to positive at the age of approximately 5 years (figure 4*e*). Variation in the estimate of the mean slope increases with population age as the number of persisting local populations decreases rapidly with increasing age.

In the empirical data of Haag *et al*. (2005), there is a similar interaction between population age and connectivity in the frequency of the *Pgi* genotype (*p*=0.013; figure 4*c*) as in the model-predicted data (figure 4*a*). Haag *et al*.'s (2005) result was based on allozymes, but there is close correspondence between the allozyme allele *f* and the nucleotide allele C in SNP AA111 in the DNA sequence (Orsini *et al*. 2009).

Turning to the level of habitat patch networks, figure 4*b* shows that, in the model-predicted data, the C allele was absent on average in 10 per cent (95% confidence limits 3.1–25%) of the patch networks due to genetic drift affecting especially the smallest metapopulations. Among the remaining networks, the frequency of C decreased with increasing metapopulation size (slope negative in 100% of the 150 independent snapshots). In the empirical data from the study of Hanski & Saccheri (2006), the C allele was absent from 27 per cent of networks, typically, again, the smallest networks, and in the remaining networks the frequency of C decreased with increasing size of the metapopulation (figure 4*d*, one-sided *p*<0.01). In the electronic supplementary material, we present comparable modelling results for a neutral locus, which does not show the patterns reported here for *Pgi*.

The results in figure 4*a*,*b* may be compared with the frequency of the C allele in a non-spatial context, in an infinitely large population with random mating, assuming the fitnesses of the different SNP AA111 genotypes given in table 1. The non-spatial model predicts an equilibrium frequency of 0.08 for the C allele (see the electronic supplementary material), maintained by the elevated fecundity of the AC heterozygotes. Both in the model predicted as well as in the empirical results (figure 4*b*,*d*), the frequency of C was generally higher than 0.08, suggesting that the elevated dispersal rate of the AC heterozygotes leads to a further advantage of the C allele in the real fragmented landscape. The AC heterozygotes have a particular advantage in the colonization of isolated habitat patches, which will be further analysed below.

### (c) *Phosphoglucose isomerase* polymorphism and demographic dynamics

Having demonstrated that the model predicts correctly two patterns in the spatial distribution of the *Pgi* genotypes, we now turn to a more detailed analysis of the influence of *Pgi* polymorphism on the ecological dynamics.

Given the assumed difference in the dispersal rate between the AA homozygotes and individuals possessing the C allele, most of which are AC heterozygotes, we would expect differences in the numbers of habitat patches visited by the different genotypes in their lifetime. The details are presented in figure S4 of the electronic supplementary material; here we summarize the main results.

As expected, there is a clear difference between the genotypes, the less dispersive AA individuals visiting on average 1.4 patches in their lifetime (including the natal patch), whereas the more dispersive AC individuals visited on average 6.6 patches, although with much variation depending on the spatial connectivity of the natal patch. Regardless of their mobility in the matrix, butterflies of both genotypes spent about the same time in the habitat patches (mean 4.9 days for AA and 5.5 days for AC). The range of lifetime movements was clearly shorter for the AA homozygotes (mean 183 m) than that for the AC and CC individuals (mean 3339 m).

**Figure 5:**
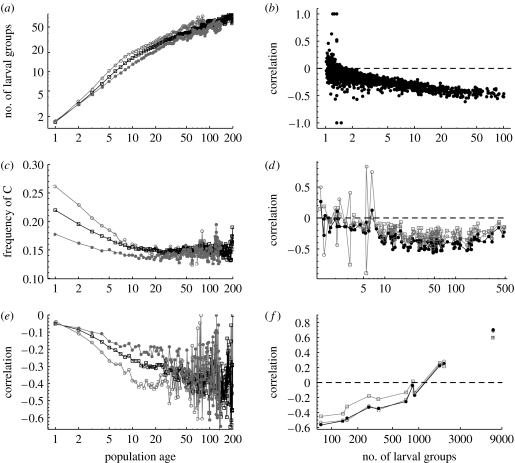
(*a*,*c*,*e*) The effect of population age on (*a*) the mean number of larval groups, (*c*) the mean frequency of the C allele and (*e*) the correlation between the number of larval groups and the frequency of the C allele in simulation results is shown. The averages in (*a*,*c*) were taken over a collection of isolated patches ($\text{ln}\;S_{i}^{\text{static}}<5.6$; open circles), well-connected patches ($\text{ln}\;S_{i}^{\text{static}}>6.2$; filled circles) and all patches (squares). The static connectivity of patch *i* is computed as $S_{i}^{\text{static}}=\sum_{j\neq i}\sqrt{A_{j}}\;\text{exp}\;(-d_{ij})$, where *A*_*j*_ is the area of patch *j* (m^2^) and *d*_*ij*_ is the distance (km) between patches *i* and *j*. The correlation in (*e*) was calculated among local populations. (*b*,*d*,*f*) The correlations between the number of larval groups and the frequency of C as a function of mean number of larval groups, at levels of (*b*) individual patches, (*d*) sub-networks of habitat patches and (*f*) large partitions of the entire Åland Islands are shown. Filled circles in (*b*,*d*,*f*) represent cross-correlations at lag of zero, open circles at a lag of less than 2 years (number of larval groups measured 2 years before the frequency of the C allele) and open squares a lag of more than 2 years (frequency of the C allele measured 2 years before the number of larval groups). In (*f*), the circles that are not joined by the curve represent the correlation at the level of the entire Åland Islands.

Following colonization, the mean number of larval groups increases and the frequency of C decreases with increasing population age, and these changes are greater in isolated than in well-connected populations (figure 5*a*,*c*). These changes generate a negative correlation between the number of larval groups and the frequency of C for all population ages, the strength of the correlation increasing with population age (figure 5*e*).

The correlation between the number of larval groups and the frequency of the C allele may vary with the spatial scale. Figure 5*b*,*d*,*f* shows the correlation as a function of the mean number of larval groups, for the spatial scales from individual habitat patches (figure 5*b*) to metapopulations in different sub-networks (figure 5*d*) and to even larger partitions of the entire Åland network (figure 5*f*). Generally, the correlations are negative, and the magnitude of the correlation increases with population size, excepting the largest partitions of the entire network, for which the correlation becomes positive. We compared the correlations obtained by recording the frequency of the C allele 2 years before (open squares) or 2 years after (open circles) recording the number of larval groups (figure 5*d*,*f*). The correlation is stronger for the latter case, suggesting that demographic dynamics may have a stronger influence on genetic dynamics than vice versa. Simulations conducted under regular patch networks were in line with the results in figure 5, the correlation between the frequency of C and population size being zero for metapopulations inhabiting sparse networks of small patches, negative for intermediate networks and positive for dense networks of large patches (see figure S5 in the electronic supplementary material).

**Figure 6:**
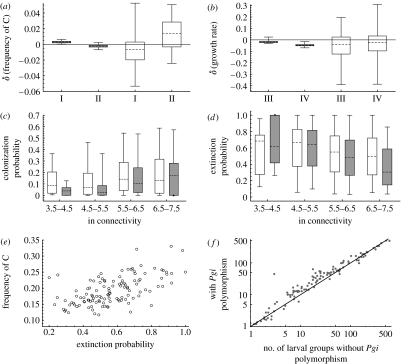
Coupling between *Pgi* polymorphism and demographic dynamics. (*a*) The genetic response to perturbation in population size (I, density is doubled; II, density is halved) is shown. (*b*) The response in the 1-year population growth rate to perturbation in the frequency of the C allele (III, frequency of C is increased; IV, frequency of C is decreased) is shown. The response is measured 1 year after the perturbation as the difference between the perturbed and unperturbed metapopulations, the variable being the frequency of C (genetic response) or the growth rate (demographic response). Simulations conducted in a 10×10 km area assuming a regular patch network with 64 patches of area 0.5 ha (white boxes, intermediate network) or 196 patches of area 1.0 ha (grey boxes, dense network). For more details on the perturbation experiment, see the electronic supplementary material. (*c*,*d*) The effects of connectivity on the probability of a patch (*c*) being colonized and (*d*) going extinct are shown. In these figures, the grey boxes represent the results without *Pgi* polymorphism, while white boxes show the results with *Pgi* polymorphism. (*e*) The mean frequency of the C allele as a function of the mean local extinction probability in a patch network is shown. (*f*) The effect of *Pgi* polymorphism on the mean number of larval groups in the autumn in each patch network level is shown, the line showing the identity *y*=*x*. The connectivity measure in (*c*,*d*) is $S_{i}^{\text{static}}$ (figure 5).

To examine the causality behind these correlations, we conducted a set of perturbation experiments (described in §3*f*) in one intermediate network with a negative correlation and in one dense network with a positive correlation (see figure S5 in the electronic supplementary material). In both cases, either increasing or decreasing the frequency of the C allele reduced the short-term growth rate (figure 6*b*), apparently because of the contrasting effects of the AC individuals having a higher growth rate but the CC individuals having reduced survival. The evolutionarily stable frequency of the C allele thus represents a compromise between these two selective forces. Turning to the demographic effects on genetic dynamics, in the intermediate network, decreasing metapopulation size leads to an increase in the frequency of the C allele (white bars in figure 6*a*) due to the superior colonization capacity of the individuals with the C allele. In the dense network (grey bars in figure 6*a*), the responses are in the opposite directions, such that increasing metapopulation size leads to an increase in the frequency of C. In this case, the AC individuals do not have an advantage in colonization as most habitat patches tend to be occupied even at relatively low density. Furthermore, the distances between the patches are short, and also the AA individuals can colonize empty patches. At high densities, density dependence becomes important, essentially truncating the population sizes to a level determined by patch size. In this situation, the AC individuals have a selective advantage, as they spread their reproductive effort among a larger number of patches, thus avoiding high mortality in the most crowded patches more effectively than the AA individuals. Finally, in dense patch networks, the overall frequency of the C allele is much lower than that in sparse networks (figure 4), and therefore increasing the frequency of the C allele does not cause much reduction in population growth due to reduced survival of the CC individuals.

Concerning the overall effect of the *Pgi* polymorphism on metapopulation dynamics, we return to figure 1, which shows the predicted long-term dynamics for the entire metapopulation in the absence and presence of *Pgi* polymorphism. This comparison suggests that the presence of *Pgi* polymorphism somewhat increases the mean metapopulation size, but does not change the amount of variability (omitting the initial 500 years; the means in figure 1*a–d* are 0.25, 6351, 0.28 and 7297, and the coefficients of variation are 0.30, 0.51, 0.32 and 0.48, respectively). In the presence of *Pgi* polymorphism, isolated patches have not only greater colonization probabilities (figure 6*c*), but also the respective populations have smaller extinction probabilities (figure 6*d*). The latter result indicates that individuals with the C allele increase the demographic rescue effect. The frequency of C increases with the mean local extinction probability in a patch network (figure 6*e*), indicating that this allele has a selective advantage when there are more opportunities for colonizing empty patches. *Pgi* polymorphism increases the metapopulation size in intermediate patch networks, but not in large patch networks (figure 6*f*).

## 5. Discussion

There are extensive theoretical (Olivieri *et al*. 1995; Bowler & Benton 2005; Ronce 2007) and empirical literatures on the evolution of dispersal, the latter especially on the evolution of wing polymorphism in insects (Roff 1986; Roff & Bradford 1996). Evolution of dispersal is receiving much attention in the context of management and conservation (Kokko & Lopez-Sepulcre 2006), since climate change, habitat fragmentation and species invasions have ecological consequences related to dispersal and its evolution. Assessing the selection gradients in the evolution of dispersal is challenging, however, as the movements of individuals are affected by complex behavioural and physiological responses to biotic and abiotic environmental conditions (Nathan *et al*. 2008), and as the evolution of dispersal is known to be affected by a large number of processes (Ronce 2007). Another important challenge, which has been the particular focus of the present work, is the feedback between the demographic dynamics and the evolutionary dynamics of dispersal-related traits.

An evolutionarily stable dispersal strategy is to make movement decisions based on information about the spatio-temporal availability of resources necessary for survival and reproduction. If there are no costs to movement and the information is complete, this leads to the ideal free distribution at the population level. Any more realistic model of the evolution of dispersal must include an explicit or implicit assumption about the cost of dispersal. An example of an implicit assumption is that individuals undergo at most one dispersal event during their lifetime, leading to the risk of moving into a location where habitat quality is lower than that in the natal population (Asmussen 1979; Myers *et al*. 1995). Explicit assumptions about the cost of dispersal include increased mortality during dispersal (Murrell *et al*. 2002; Rousset & Gandon 2002), the related assumption about distance-dependent dispersal (Heino & Hanski 2001) and time lost during dispersal. Our model includes the latter two assumptions. Glanville fritillary females typically spend much time searching for oviposition host plants before accepting one (Singer & Hanski 2004). There is presently no quantitative information on the extent to which time spent during dispersal reduces the oviposition rate, and we have made the somewhat simplistic assumption that the realized oviposition rate is proportional to the search time.

Predictive modelling of the evolution of dispersal calls not only for realistic movement models but also for knowledge about the trade-offs that may involve dispersal traits. Dispersal ability is often observed or assumed to trade off with the other life-history traits, so that the most dispersive individuals have reduced fecundity, survival or competitive ability (e.g. wing dimorphic insects, Roff & Bradford 1996). Somewhat unexpectedly, there is no trade-off between dispersal rate and ovipositing rate or even lifetime fecundity, in the Glanville fritillary (Hanski *et al*. 2006; Saastamoinen 2007*b*). On the other hand, there are other trade-offs at molecular, physiological and population levels that are potentially important.

A trade-off involving molecular and physiological traits, although not included in our model, relates to an interaction between ambient temperature and *Pgi* genotype in affecting dispersal rate. The difference in the flight metabolic rate and the observed dispersal rate in the field between the AC and AA genotypes is the greatest in low ambient temperatures (Niitepõld *et al*. in press), probably because of temperature-dependent enzyme kinetics between the different isoforms of the *Pgi* enzyme (Watt 1983, 1992). We based our assumptions about the genotypic difference in dispersal on measurements in low-temperature conditions, because such conditions are frequent at the high latitude where the study metapopulation is located.

The highly dispersive AC individuals are good colonizers, but at the same time they exhibit a high rate of emigration from habitat patches, and, therefore, they often disperse away from high-quality patches sooner than would be optimal. This relationship between immigration and emigration can be interpreted as a population-level trade-off (Hanski *et al*. 2006). On the other hand, fast emigration has the benefit of reducing inbreeding. Butterflies that eclose in the generally small newly established populations are likely to mate with their full sibs, selecting against the good dispersers with the AC genotype because of the low fitness of the CC homozygotes. Inbreeding depression generally selects for elevated dispersal (Bengtsson 1978; Waser *et al*. 1986; Motro 1991; Gandon 1999).

Owing to the difficulties in obtaining relevant data for quantitative modelling of evolution of dispersal, there has been limited success in using models to make quantitative predictions and testing these predictions against empirical data. In the present work, we have attempted to bridge the gap between theory and empirical work by using the large amount of empirical information that is available for the Glanville fritillary butterfly to construct a realistic individual-based model. Owing to the complexity of the model, and the need to adjust some parameters to match the predicted and observed demographic dynamics, we cannot test the predicted ecological dynamics with independent data. On the other hand, we could test two predictions about the spatial variation in the *Pgi* allele frequency among individual populations and among independent metapopulations occurring in sub-networks with different numbers, sizes and connectivities of habitat patches (figure 4). The model correctly predicts increasing frequency of the *Pgi* SNP AA111 C allele with decreasing connectivity in newly established populations, and decreasing frequency with decreasing connectivity in old populations. At the patch network level, the frequency of C was predicted and observed to increase with decreasing metapopulation size. This result is in line with the general expectation that high rate of local extinction, which is the case for small metapopulations occupying marginal patch networks, generally selects for increased dispersal rate in providing more opportunities for re-colonization (Heino & Hanski 2001; for a review see Ronce & Olivieri 2004).

Previous observational studies on the large metapopulation of the Glanville fritillary butterfly have strongly suggested that demographic dynamics and evolution of dispersal affect each other (Hanski & Saccheri 2006; Saccheri & Hanski 2006). Our results provide detailed insight into the nature of this coupling. The demographic dynamics impose a number of selection pressures at the *Pgi* locus. Elevated population turnover rate in highly fragmented landscapes selects for the C allele, especially when the metapopulation is at low density and there are opportunities for colonization. In less fragmented landscapes, the more dispersive genotype is selected for when the metapopulation reaches high density, probably because in these situations the level of resource and kin competition become elevated, selecting for more dispersal (Hamilton & May 1977; Frank 1986; Taylor 1988). Thus, variation in demographic dynamics creates variation in selection gradients, which helps maintain *Pgi* polymorphism at the regional level.

Considering the genetic effects on demographic dynamics, the *Pgi* polymorphism apparently helps the species persist in marginal situations, due to more effective colonization of unoccupied habitat (by the AC heterozygotes) and rapid use of the respective resources (by the AA homozygotes). As selection operates at the level of individuals, evolution does not optimize the fitness or size of a population (Hamilton & May 1977; Comins *et al*. 1980). In extreme cases, the mismatch between the ‘best for the individual’ and the ‘best for the population’ may lead to an evolutionary suicide (Gyllenberg *et al*. 2002). However, our perturbation experiments with the present model suggested that under several network structures, the allele frequencies in the *Pgi* gene evolve to values that are close to the optimal in terms of demographic performance, as perturbing the *Pgi* allele frequency either upwards or downwards from the evolved value led to reduced growth rate. By contrast, perturbing the metapopulation size upwards or downwards leads to opposite responses in the *Pgi* allele frequency, the directions of these responses depending further on the size of the metapopulation. In brief, there is a strong coupling between the ecological metapopulation dynamics and the genetic dynamics in a locus affecting dispersal and other life-history traits in this system.

## Figures and Tables

**Table 1:** Parameter values of the individual-based model.

parameter	value	explanation, unit
*movement phase*		
*D*_m_	500 000	diffusion coefficient in the matrix for the monomorphic population
$D_{\text{m}}^{\text{H}}$	5 000 000	diffusion coefficient in the matrix for genotypes AC and CC, m^2^ d^−1^
$D_{\text{m}}^{\text{L}}$	50 000	diffusion coefficient in the matrix for genotype AA, m^2^ d^−1^
*D*_p_	140	diffusion coefficient in patches, m^2^ d^−1^
*k*_p_	400	preference for patches for the monomorphic population
$k_{\text{p}}^{\text{H}}$	1300	preference for patches for genotypes AC and CC
$k_{\text{p}}^{\text{L}}$	130	preference for patches for genotype AA
*t*_c_	1	time spent in the natal patch before starting the movement mode, d
*μ*_p_	0.14	mortality in patches, d^−1^
*μ*_m_	0.14	mortality in matrix, d^−1^
*η*_0_	0.02	tendency for multiple mating
*λ*_M_	1	rate of mating, d^−1^
*λ*_L_	0.4	rate of laying egg clutches, d^−1^
*larval survival*		
*μ*_s_	logit(0.27)	mean survival rate of larval groups over winter
*σ*_s_	1.0	standard deviation of winter survival
*d*_c_	10 000	average distance of spatial autocorrelation in survival rate of larval groups over winter, m
*e*_*p*_	0.05	probability of all larval groups in a patch going extinct
*n*_0_	6	number of adults that would hatch per larval group in the absence of density dependence
*c*_0_	25	prefactor in the carrying capacity $K_{i}=n_{0}c_{0}A_{i}^{c_{1}}$, where area unit is ha
*c*_1_	0.32	exponent in the carrying capacity
*genetics*		
*f*_1_	0.2	fraction of increased fecundity when the female is AC
*f*_2_	0.9	probability of death before eclosion for genotype CC
*u*	10^−6^	mutation rate per locus per generation for allozyme loci
*u*	10^−3^	mutation rate per locus per generation for microsatellite loci
*N*_*l*_	(12, 4, 3, 3, 3, 9, 11)	number of alleles at neutral locus *l*

**Table 2:** Sensitivity of habitat patch occupancy, total number of larval groups in the metapopulation and population turnover to dispersal rate in the matrix *D*_m_, habitat selection *k*_p_ and nest survival rate over winter logit^−1^(*μ*_s_). (For each combination of parameters, we simulate neutral ecological dynamics with parameter values shown in table 1. The quantile 0.5 (quantiles 0.025, 0.975) of the fraction of occupied patches (occupancy), number of larval groups, number of colonizations and number of extinctions were calculated for the autumn population stage and the initial 500 years were dropped if the population persisted up to 1000 years.)

*D*_m_ (m^2^ d^−1^)	*k*_p_	logit^−1^(*μ*_s_)	years persisted	occupancy	no. of larval groups	no. of colonizations	no. of extinctions
5×10^4^	130	0.2	26	0.03 (0, 0.42)	767 (1, 17 042)	19 (0, 555)	29 (0, 434)
		0.25	43	0.03 (0, 0.19)	840 (11, 4866)	22 (2, 101)	25 (3, 187)
		0.3	559	0.04 (0.01, 0.16)	1473 (162, 5658)	9 (1, 24)	10 (0, 39)
5×10^5^	400	0.2	30	0.03 (0, 0.54)	316 (3, 15 975)	43 (1, 856)	44 (6, 495)
		0.25	212	0.08 (0.01, 0.26)	1111 (26, 6956)	70 (7, 222)	67 (14, 274)
		0.3	1000	0.36 (0.23, 0.49)	10 024 (4466, 18 384)	184 (119, 266)	179 (106, 296)
5×10^6^	1300	0.2	500	0.18 (0, 0.57)	2046 (5, 13 161)	122 (3, 272)	132 (3, 347)
		0.25	1000	0.52 (0.37, 0.63)	13 857 (5223, 26 691)	204 (131, 288)	200 (123, 318)
		0.3	1000	0.62 (0.52, 0.68)	23 233 (11681, 35 901)	180 (123, 256)	176 (115, 263)

## Supplementary Material

Detailed description of the simulation model Evolutionary model of dispersal for the glanville fritillary butterfly
