# Supplementary material for: Modelling single nucleotide effects in phosphoglucose isomerase on dispersal in the Glanville fritillary butterfly: coupling of ecological and evolutionary dynamics
Source: Philos Trans R Soc Lond B Biol Sci. 2009 Jun 12;364(1523):1519–32. doi: 10.1098/rstb.2009.0005 (PMC2690501; doi:10.1098/rstb.2009.0005)
Supplement: Detailed description of the simulation model — Evolutionary model of dispersal for the glanville fritillary butterfly [file rstb20090005s04.pdf]

Supplementary material: Detailed description of the simulation model

Simulating butterfly movements

Movements of adult butterflies were simulated using the diffusion approximation of a random walk model with habitat selection. We base the simulation on the analytic expressions for the quantities $P_{ij}, F_{i|j}, F_{i|0}, G_{i|j}, G_{i|0}$ derived by Ovaskainen and Cornell (2003) and Zheng et al. (2009). P_{ij} denotes the probability that an individual initially in patch *i* arrives at patch *j* before it dies or arrives at any other patch. $F_{i|j}\left(F_{i|0}\right)$ denotes the time that an individual initially in patch *i* is expected to spend in patch *i* conditional on that it next moves to patch *j* (or dies before moving to any other patch). $G_{i|j}\left(G_{i|0}\right)$ denotes the time that an individual initially in patch *i* is expected to spend in the matrix conditional on that it next moves to patch *j* (or dies).

These quantities were applied as follows. Assume that an individual has just arrived at (or eclosed in) patch *i*. To simulate the subsequent movements of this individual, we first use the probabilities P_{ij} to randomize the patch to which the individual would move next (or die). We then compute the mean time $F_{i|j}\left(F_{i|0}\right)$ that the individual was expected to spend in patch *i* before emigrating (or dying), and the mean time $G_{i|j}\left(G_{i|0}\right)$ the individual was expected to spend in the matrix before immigrating to patch *j* (or dying). The realized times were assumed to follow an exponential distribution with these means. The simulation yields individual movement histories in continuous time, with location classified as the individual being in a given patch or in the matrix.

Parameter values

Movement behavior

Harrison et al. (in prep.) have estimated the parameters of the diffusion model for the Glanville fritillary using the method of (Ovaskainen et al. 2008b) and the data described by Hanski et al. (1994). These data allow the estimation of diffusion parameters separately for the habitat patches (based on one large and well-studied population; 1994) and the landscape matrix. For the monomorphic population we used the average values for the two sexes: D_m =

5×10^5, D_p =140, k_p=400, and the mortality rate (per day) during dispersal μ=0.14. Parameter values for the different *Pgi* genotypes cannot be estimated rigorously, but the values in Table 1 in the main text are consistent with the results of K. Niitepõld et al. (2008), who report a large difference between the genotypes in dispersal rate in the field. There is a significant interaction with ambient temperature, such that the difference between the genotypes is greatest in lower temperatures (however, we do not attempt to model the temperature effect). The value of t_c=1 day (the time spent in the natal patch before starting the dispersal phase) is based on Ovaskainen et al. (2008b).

Mating and oviposition

The parameter values relating to mating and oviposition are based on extensive data from experiments conducted in a large outdoor population cage (Hanski et al. 2006, Saastamoinen 2007a, b). The incidence of multiple mating depends on population density and environmental conditions. The value used of Table 1 is consistent with our observations but is not critical to the results.

Larval survival

Survival of larval groups over winter varies from year to year, from 50 to 84% (Nieminen et al. 2004). The average value and standard deviation in Table 1 have been adjusted to roughly match the model-predicted patch occupancy in the metapopulation with the empirical results. The scale of spatial autocorrelation (d_c) is consistent with the results of Hanski and Meyke (2005). Two parameters of local dynamics, n_0 and λ_L, are consistent with the results of Kuussaari et al. (2004) and Hanski et al. (1994), but the values have been adjusted to match the predicted level of patch occupancy and the number of larval groups per population with the observed data. The prefactor c_0 was set to yield the carrying capacity of 150 for a patch of 1 ha (Hanski et al. 1994), and the scaling parameter c_1 was estimated by regressing the logarithm of the number of larval groups against the logarithm of patch area in the pooled empirical data from the entire Åland Islands for 1993 to 2006.

Scaling between the parameters D and k

We assume that individuals with the *C* allele in *Pgi* SNP AA111 are more mobile (high mobility, denoted here by H) than individuals without the *C* allele (low mobility, denoted here by L). In the context of a diffusion model, greater mobility generally corresponds to a larger diffusion coefficient. As the diffusion coefficient integrates over several characteristics of the movement behaviour, including step length and the frequency at which the steps are taken, there are several possible mechanisms that may generate the difference in the diffusion coefficient between the L and H individuals. As discussed below, these assumptions can lead to a dissimilar scaling between the diffusion coefficient D and the parameter k, which measures the ratio between the density of individuals in the habitat patches over the density of individuals in the matrix.

We assume that individuals move in discrete hops, with a given distribution of step lengths and movement frequencies. As the shapes of these distributions (or the distribution of turning angles, assuming that step lengths and movement frequencies are the same for L and H individuals) do not alter our calculations below, we assume for simplicity that there is a constant step length and a constant movement frequency. The values of these parameters are assumed to be specific to the type of individuals (L or H) and to the habitat type (patch or matrix). Assume that the L and H individuals are otherwise identical, except that

- Case I: The step length for H individuals is x times greater than for L individuals, both in the patches and in the matrix.
- Case II: The movement frequency for H individuals is x times greater than for L individuals, both in the patches and in the matrix.
- Case III: The step length for H individuals is x times greater than for L individuals in the matrix. The step length in the patches is the same for H and L individuals.
- Case IV: The movement frequency for H individuals is x times greater than for L individuals in the matrix. The movement frequency in the patches is the same for H and L individuals.

The diffusion coefficient scales as $D \propto \lambda^2 / \tau$, where λ is the step length and τ is the waiting time between the steps. The movement speed s scales as $s \propto \lambda / \tau$. Normalizing the density in the matrix to 1, the density in the patches scales as $k_p \propto b(s_m / s_p)$, where b is a behavioural parameter measuring the probability by which an individual turns back after crossing the patch boundary (Ovaskainen and Cornell 2003). Assuming that the H and L individuals have

the same behavioural parameter *b*, we have $k_p^H = k_p^L$ in Cases I and II, $k_p^H = k_p^L \sqrt{D_m^H / D_m^L}$ in Case III, and $k_p^H = k_p^L (D_m^H / D_m^L)$ in Case IV. Assuming a 100-fold difference in the matrix-specific diffusion rate between the H and L individuals, we obtain the numerical values shown in Table S1. Note that there is no difference between Cases I and II.

Table S1. Numeric values of movement parameters consistent with the assumptions described for Cases I-IV.

	D_p^L	D_p^H	D_m^L	D_m^H	k_p^L	k_p^H
Case I	14	1,400	50,000	5,000,000	400	400
Case II	14	1,400	50,000	5,000,000	400	400
Case III	140	140	50,000	5,000,000	130	1300
Case IV	140	140	50,000	5,000,000	40	4000

We have no detailed information on the movement behaviour of the H and L individuals that could be used to distinguish between the above four cases. We decided to use parameters consistent with Case III for the following reasons. First, in Cases I and II, the H individuals emigrate so fast from the habitat patches that they end up spending most of their time in the matrix (Fig. S1). This would give a great selective advantage for the L individuals (recall that egg-laying is proportional to the time spent in patches), and thus lead to a much lower frequency of the *C* allele than observed in the empirical data. In Cases III and IV, the H and L individuals behave similarly within the patches and have thus the same emigration rate (Figs. S2 and S3). In Case IV, the H individuals are very effective dispersers, moving through the matrix so fast that they end up spending much more time in the habitat patches than the L individuals (Fig. S3). The Case III represents an intermediate case in which the H and L individuals spend roughly an equal amount of time in the habitat patches (Fig. S2), not giving either one a major selective advantage due to a difference in the time spent in the matrix.

Supplementary results

Equilibrium allele frequencies in a non-spatial context

Consider a non-spatial model for a single population with infinite size and random mating. According to the table below, the recursive relations for the genotypes *AA*, *AC* and *CC* (labeled as *P*, *Q* and *R*) are given by

$$P' = \frac{P*}{P*+Q*+R*},$$

$$Q' = \frac{Q*}{P*+Q*+R*},$$

$$R' = \frac{R*}{P*+Q*+R*},$$

where

$$P* = P^2 + 0.5PQ + 0.5(1+f_1)QP + 0.25(1+f_1)Q^2,$$

$$Q* = 0.5PQ + 2PR + 0.5(1+f_1)(QP + Q^2 + QR) + 0.5RQ,$$

$$R* = 0.25(1+f_1)(1-f_2)Q^2 + 0.5(1+f_1)(1-f_2)QR + 0.5(1-f_2)RQ + R^2(1-f_2).$$

Here the values with the prime denote frequencies in the next generation, f_1 is the proportional increase in fecundity if the genotype of the female is *AC*, and f_2 is the proportion of *CC* individuals that die before eclosion. Setting f_1=0.2 and f_2=0.9, and solving the recursive relations numerically, we obtain the equilibrium values *P*=0.834, *Q*=0.165, and *R*=0.000814. Thus the equilibrium solutions for the frequencies of alleles *A* and *C* are 0.917 and 0.083, respectively.

Table S2. Relative numbers of adults developing from a larval group laid by parents of a given combination of genotypes.

Mother × Father	Frequency	Offspring			
		AA	AC	CC	Sum
AA × AA	P^2	1	0	0	1
AA × AC	PQ	0.5	0.5	0	1
AA × CC	PR	0	1	0	1
AC × AA	QP	0.5 (1+f_1)	0.5 (1+f_1)	0	1+f_1
AC × AC	Q^2	0.25 (1+f_1)	0.5 (1+f_1)	0.25 (1+f_1) (1-f_2)	0.25 (1+f_1) (4-f_2)
AC × CC	QR	0	0.5 (1+f_1)	0.5 (1+f_1) (1-f_2)	0.5 (1+f_1) (2-f_2)
CC × AA	RP	0	1	0	1
CC × AC	RQ	0	0.5	0.5 (1-f_2)	1-0.5 f_2
CC × CC	R^2	0	0	1-f_2	1-f_2

Dynamics in a neutral locus in relation to patch age and connectivity

We repeated the analysis of Figs. 4a, b assuming a neutral microsatellite locus with 9 alleles. We labelled two of the alleles as the *C* allele and the remaining 7 alleles as the *A* allele. This gives the frequency of 2/9 for the hypothetical *C* allele, comparable to the overall mean frequency of the real *C* allele in *Pgi* SNP AA111. Assuming neutrality, the probability of obtaining a smaller slope for the new populations than for the old populations in Fig. 4a would be 0.55. In the analysis in Fig. 4b, the mean fraction of networks in which the *C* allele is absent was 57% (95% confidence intervals 8.4%-94%), and the regression slope for the remaining networks was negative in 40% of the simulations. These results illustrate that the patterns of Figs. 4a, b cannot be explained by genetic drift.

Movement behaviour of H and L individuals

Figure S2 shows movement statistics of the diffusion model for individuals with and without the *C* allele, assuming that the focal individual is initially in a patch with the area given on the horizontal axis. These results are based on the analytical formulae, and they are shown for a single patch network. In Fig. S4 we show comparable results based on the simulation model run over the entire Åland Islands, in which the individuals spend extra time in their natal patch before they start obeying the diffusion model. Note that here we show the realized times instead of the expected times, explaining the greater variability as compared to Fig. S2.

Coupling between demographic and genetic dynamics

Figure 5 shows how the correlation between the frequency of the *C* allele and population size depends on the metapopulation size, the comparison being done over the heterogeneous patch networks of the Åland Islands. To examine how these results depend on the density of patches (number per unit area) and on the sizes of the patches, we repeated the simulations in hypothetical patch network consisting of identical patches arranged as a regular grid within an area of 10 km x 10 km. Figure S5 shows that the correlation between the frequency of the *C* allele and metapopulation size is zero for the smallest networks, negative for intermediate networks, and positive for the largest networks, consistent with the result in Fig. 5 in the main text for the real patch networks in the Åland Islands.

Figure legends for the supplement

Fig. S1. Dispersal statistics for Cases I and II (see *Scaling between the parameters D and k*). The left-hand panels correspond to the individuals with low mobility, the right-hand panels to individuals with high mobility. Panels (a, b) show mean of the total time that the individuals are expected to spend in the habitat patches, the line corresponding to the expected lifetime. Panels (c, d) show the mean time spent in the current patch before dying or migrating to another patch, panels (e, f) the mean number of patches visited (including the natal patch), and panels (g, h) the probability of moving to another patch before dying. Results are shown for one representative sub-network of habitat patches in the Åland Islands.

Fig. S2. As Fig. S1 but for Case III.

Fig. S3. As Fig. S1 but for Case IV.

Fig. S4. A representative snapshot from the model simulations describing the dispersal phase for the individuals without (left-hand panels) and with (right-hand panels) the *C* allele. Each point gives the average over individuals that eclosed in a particular habitat patch, plotted against the area of that patch. The panels give the total time spent in the habitat patches (a, b), the number of patches visited during life-time (c, d), and the range of life-time movements, measured as the maximum distance between the patches visited and the natal patch (e, f).

Fig. S5. Correlation between population size and the frequency of the *C* allele in regular patch networks located within an area of 10 km $\times$10 km. The x-axis gives the number of patches, the y-axes the area of each patch. The networks shown by dots are analyzed further in Fig. 6 in the main text.

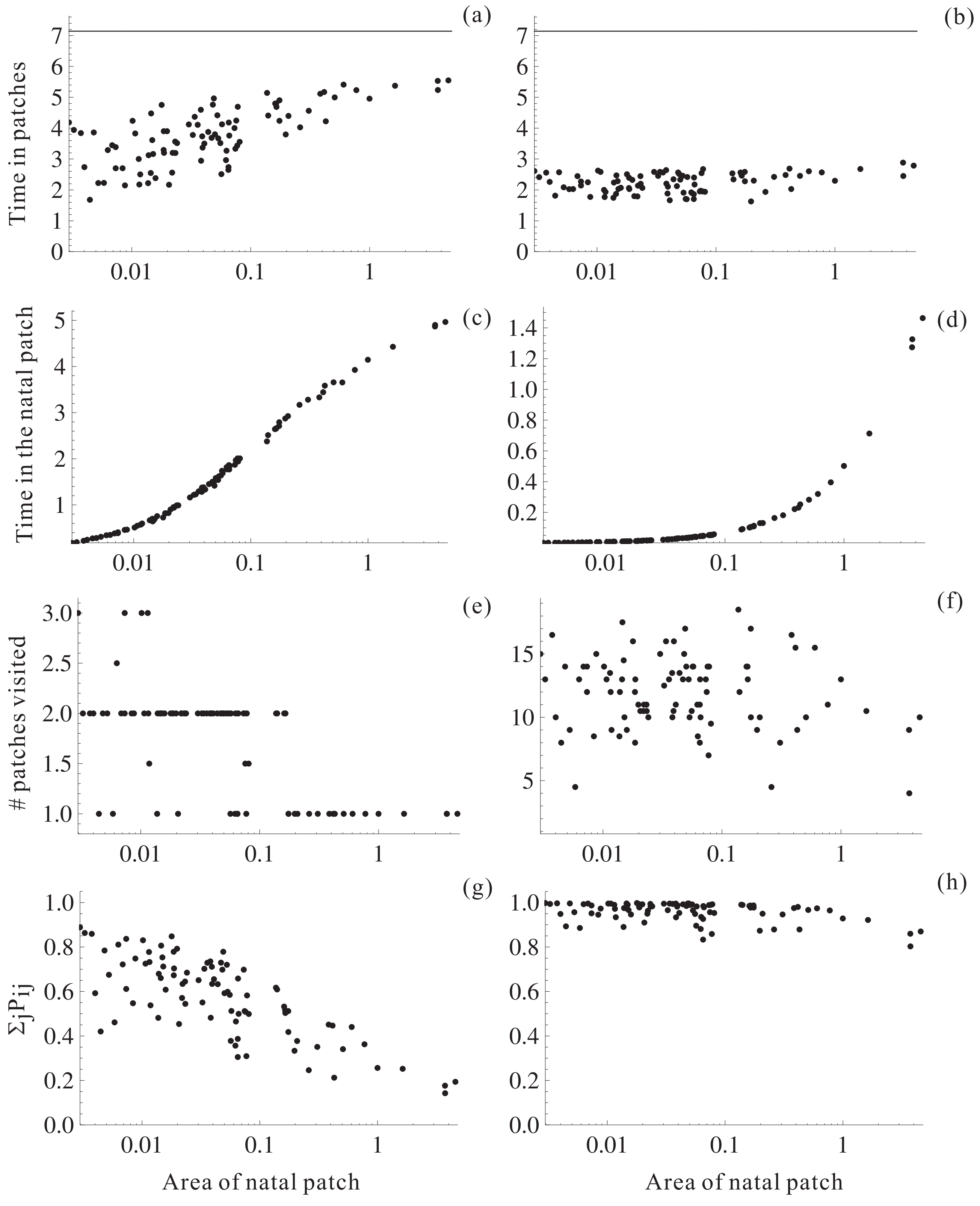
(a)
(b)
(c)
(d)
(e)
(f)
(g)
(h)
Time in patches
Time in the natal patch
patches visited
$\Sigma_j P_{ij}$
0.01
0.1
1
Area of natal patch
Area of natal patch

FIGURE S1

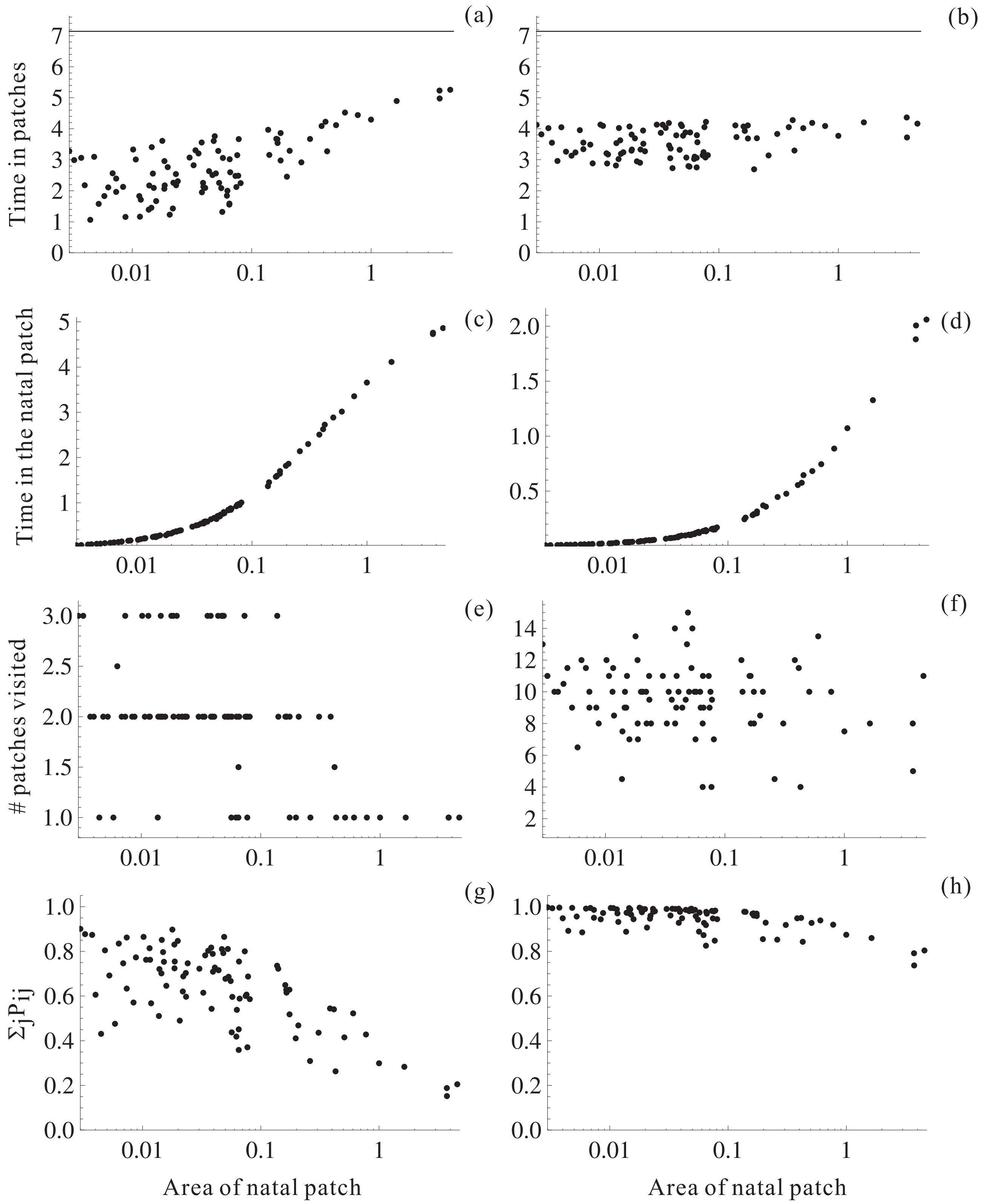
(a)
(b)
Time in patches
(c)
(d)
Time in the natal patch
(e)
(f)
patches visited
(g)
(h)
$\Sigma_j P_{ij}$
Area of natal patch
Area of natal patch

FIGURE S2

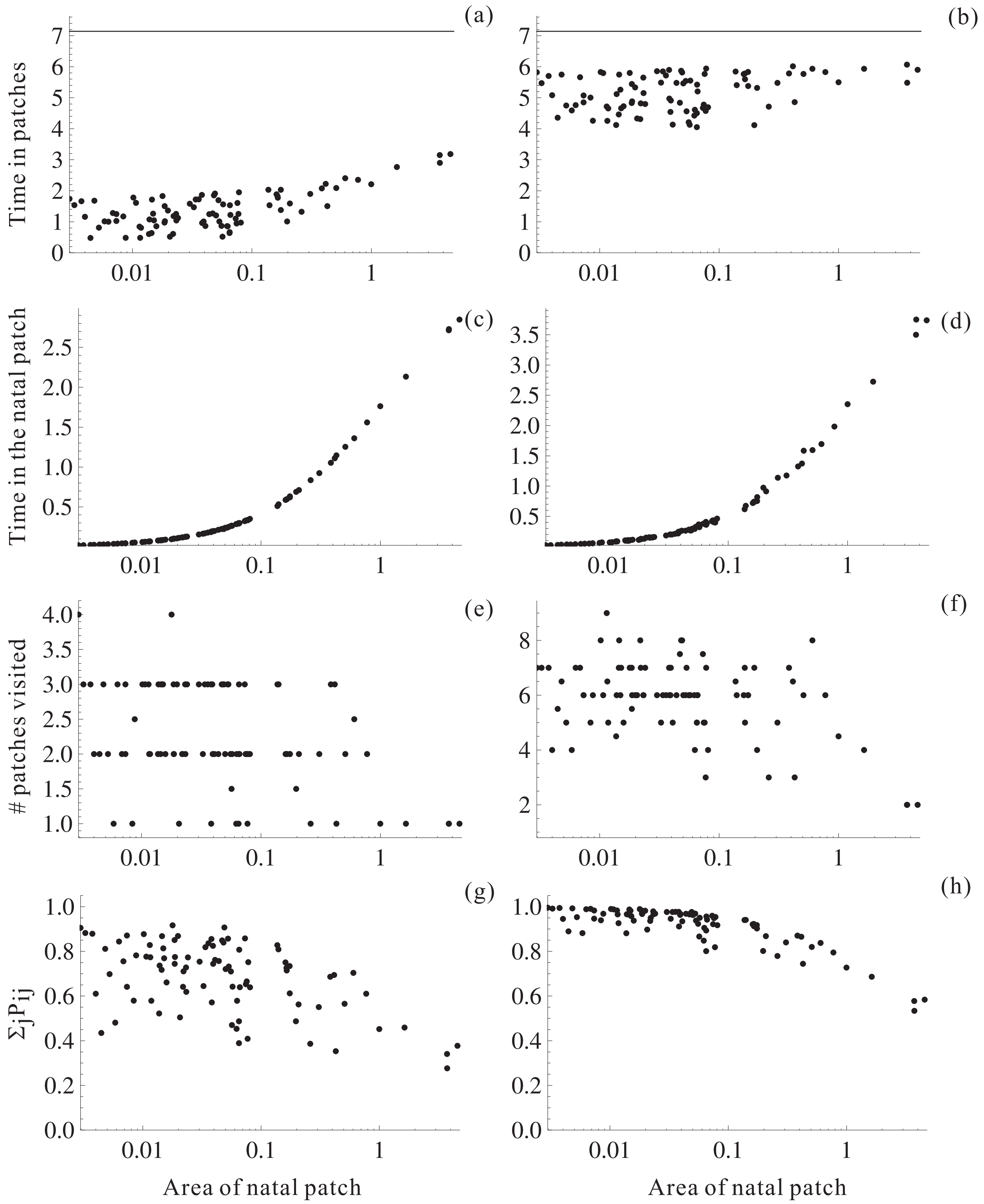
(a)
(b)
(c)
(d)
(e)
(f)
(g)
(h)
Time in patches
Time in the natal patch
patches visited
ΣjPij
Area of natal patch
Area of natal patch

FIGURE S3

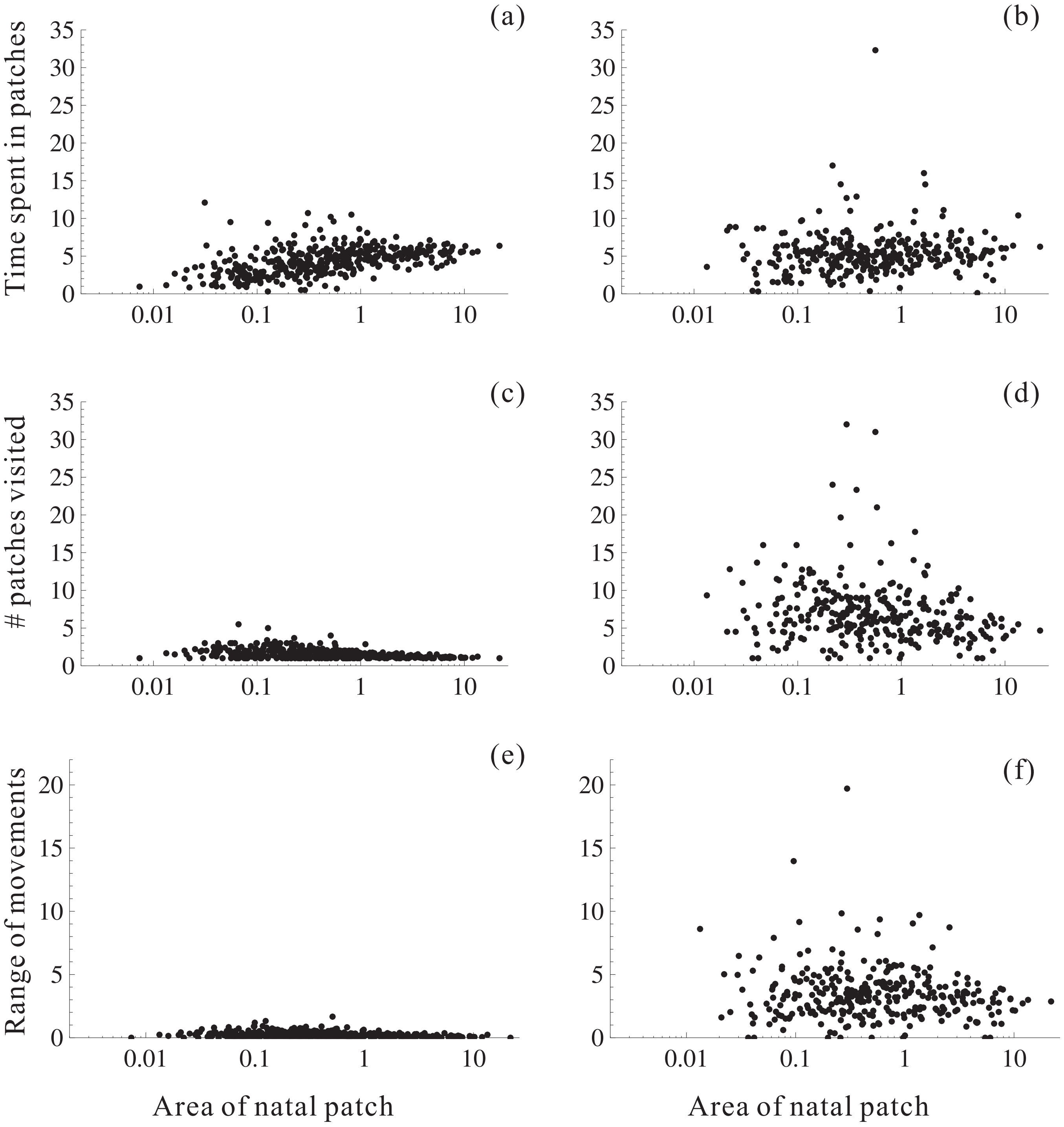
(a)
(b)
(c)
(d)
(e)
(f)
Time spent in patches
patches visited
Range of movements
Area of natal patch
Area of natal patch
0
5
10
15
20
25
30
35
0.01
0.1
1
10

FIGURE S4

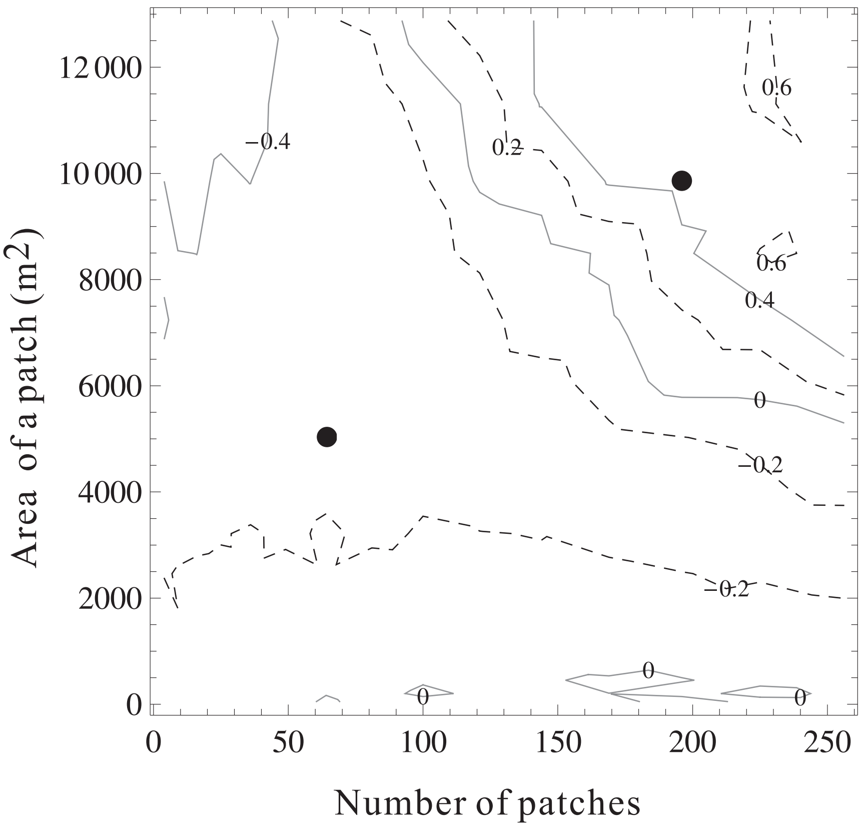

12 000
10 000
8000
6000
4000
2000
0
Area of a patch (m2)
0
50
100
150
200
250
Number of patches
−0.4
0.2
0.6
0.6
0.4
0
−0.2
−0.2
0
0
0

FIGURE S5
